# Tissue reaction to novel customized calcium silicate cement based dental implants. A pilot study in the dog

**DOI:** 10.1007/s10856-021-06512-y

**Published:** 2021-05-22

**Authors:** Amir Fakhrzadeh, Mohammad Ali Saghiri, Steven M. Morgano, Andrew Sullivan

**Affiliations:** 1 Department of Periodontics, Rutgers School of Dental Medicine, Newark, NJ USA; 2 Department of Restorative Dentistry, Rutgers School of Dental Medicine, Newark, NJ USA; 3 Department of Endodontics, University of the Pacific, Arthur A. Dugoni School of Dentistry, San Francisco, CA USA

## Abstract

**Objectives:**

The purpose of this study was to determine the level of periodontal tissue regeneration in a canine model following post-extraction placement of an implant molded from a composite material made from extracted tooth dentin and a calcium silicate cement (CSC) material. The investigation used autologous dentin in conjunction with a CSC material to form a composite implant designed for immediate tooth replacement.

**Methods:**

Two (2) beagles had a periodontal and radiographic examination performed to rule out any pre-treatment inflammation, significant periodontal disease, or mobility. Then, ination eleven (11) teeth were extracted and polyvinyl siloxane molds were made to fabricate three different types of implants: Particulate Implant (Test Group 1, *n* = 4), Shell Implant Alone (Test Group 2, *n* = 2), Shell Implant with Emdogain® (Test Group 3, *n* = 3). Teeth in the control group were extracted, scaled (*n* = 2), and then re-implanted into their respective fresh extraction sockets. At 4 weeks, a clinical, radiographic, and histologic assessment was performed.

**Results:**

Clinical evaluation revealed no mobility in any of the test or control implants and no radiographic evidence of significant bone loss or active disease. Based on the MicroCT analysis, direct bone to implant contact was observed in some areas with an apparent periodontal ligament space. Implant-related inflammation, on average, was similar among all groups, with low numbers of infiltrates. Implant-related inflammatory reaction was generally minimal and not interpreted to be adverse.

**Conclusion:**

The proposed novel composite materials revealed that not only do these materials demonstrate high biocompatibility, but also their successful integration in the alveolus is likely secondary to a partial ligamentous attachment. The current investigation may lead to the use of calcium silicate-based materials as custom dental implants. Further research on this novel composite’s biomechanical properties is necessary to develop the optimal material composition for use as a load-bearing dental implant.

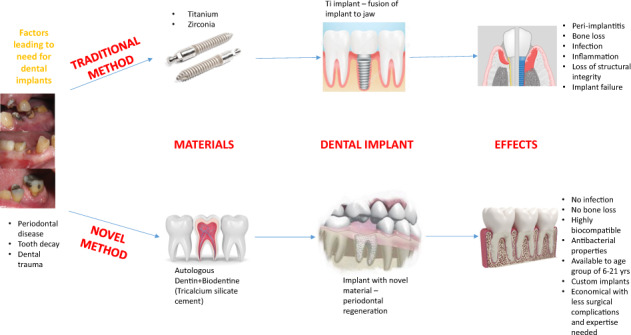

## Introduction

Titanium screw-type dental implants are the current standard for replacing failing teeth that require extraction because of dental caries, periodontal disease, or trauma. The dental implant effectively replaces natural root function by connecting to both the jawbone and the crown via an abutment. In most cases, teeth are extracted, the socket is filled with some bone void filler, and the implant is placed 4–6 months later after the bone graft has healed. After the implant is placed, an additional 3–6 months is generally required for healing before the implant can be loaded (restored with an abutment and crown) functionally, which requires several appointments for the final restoration. The typical process for placing standard dental implants involves a surgical procedure where a hole is drilled into the bone and the implant, typically made of titanium alloy (or other metal or ceramic material), is inserted (or threaded) into the cavity and allowed to fuse with the bone by a process known as osseointegration [1]. While dental implants are the current state of the art for tooth replacement, they involve an invasive surgical procedure and significant risks. Failures can occur because of prosthetic material failure, improper surgical placement, resulting in damage to adjacent teeth or vital anatomical structures, poor esthetics, infection, and disease affecting the supporting bone to implant interface known as peri-implantitis [2]. Peri-implantitis is becoming a significantly more prevalent issue because of the increased number of implants that are failing over time [3]. Often when an implant fails, the amount of bone already lost or the amount that must be removed to retrieve the titanium implant can be catastrophic, and this loss of bone makes replacing that implant extremely difficult, if not impossible, in some cases [4]. In addition, in the case of immediate implants (implant placement at the time of extraction), the defects found in extraction sockets are generally grossly different from the dimensions of the screw-type implant. Thus, primary stability and favorable placement of the implant in the bone can be challenging to achieve. This issue makes traditional implant placement at the time of extraction a far more complex procedure, increasing the risk of surgical complications, and it often is simply impossible to perform adequately.

What our group at Rutgers University has developed is a novel method and composite material for the application of immediate tooth replacement. This method involves removing the patient’s failing tooth, and as opposed to discarding it, the dentin is processed into either a particle or shell form, sterilized, and then reconstructed into a tooth form implant that mimics the shape and composition of the original tooth. This strategy combines traditional techniques used in dentistry such as tooth reimplantation with orthodontic splinting and currently emerging digital dentistry technologies to provide patients with an alternative to traditional implant surgery. Commercially available calcium silicate-based cement (CSC) material was used (Biodentine®), which has shown great promise in other dental applications and yields a composite material, which is much closer in composition, appearance, and mechanical properties to natural teeth. This material, Biodentine®, manufactured by Septodont® (Saint-Maur-des-Fossés, France), is a derivative of the traditional mineral trioxide aggregate (MTA®) cements that have been used extensively in our field because of their promising regenerative, antibacterial, and adhesive properties in and around teeth. In endodontic, periodontal, and restorative cases, the use of Biodentine® cement has demonstrated the ability to stimulate the formation of not only alveolar bone, but also a new periodontal (cementum, PDL, and bone) attachment at its interface [5]. Numerous studies have shown its ability to induce the release of pro-osteogenic factors and markers such as osteopontin, alkaline phosphatase (ALP), pyrophosphatase, bone morphogenetic protein (BMP)2, and transforming growth factor (TGF)- ß1, thereby increasing the proliferation and mineralization of osteoblasts, cementoblasts, and odontoblasts [6, 7]. This bioactive cement mimics the mechanical properties of natural teeth in terms of material strength and high biocompatibility, and it has been marketed as a dentin substitute with favorable long-term mechanical and antibacterial properties.

Literature regarding the application of CSC for pulp with capping [8–10], we anticipate that this cement composite can have excellent biocompatibility. In addition, CSCs have a very high alkaline pH, which is responsible for their antibacterial effect. The release of Ca ions is one of the main regenerative features of CSCs that induce the formation of tertiary dentin [11]. The goal is to investigate how the processing and sterilization of autologous dentin can be used in conjunction with a regenerative cement material to form a composite that, when implanted, mitigates the risk of surgical implant complications and accomplishes the goal of periodontal tissue regeneration around these dental implants.

## Materials and methods

The animal study protocol was approved by the NAMSA Northwood Division Institutional Animal Care and Use Committee (IACUC) by protocol #19-06 LLT. The study included 2 young adult male beagles, and all surgical procedures were performed under general anesthesia by a NAMSA veterinarian. Once induced, the animals were then intubated, and 0.5% bupivacaine local anesthesia was administered. Full mouth supragingival scaling and prophylaxis were performed. Before extractions, a periodontal examination was performed, and tooth mobility was scored based on the Grace & Smales Mobility Index. Teeth were elevated and delivered with forceps in an atraumatic fashion. The implants, which came from two canines, and included native tooth controls (teeth extracted, scaled, and re-implanted), implant controls (“T0,” non-implanted implants), and three treatment groups. The treatment groups included molded composites which were made of: (1) dentin powder resulting from grinding the entire tooth, mixed with dental cement (“T1”); (2) dentin shells resulting from grinding only the internal portion of the tooth, mixed with dental cement (“T2”); and (3) dentin shells resulting from grinding only the internal portion of the tooth, mixed with dental cement, and inserted along with Emdogain® (“T3”). The as-received tissues and “T0” implants were scanned and reconstructed by using microCT analyses. All control and test groups included by one maxillary and one mandibular implant limited to the anterior (incisor) or posterior (first premolar) sites. For the Control Group (No Treatment, C1): Teeth were extracted, the roots were scaled to remove any calculus or residual cementum and periodontal ligament fibers (using a scaler and rotary instrument), held extra-orally in sterile saline (or the animal’s saliva) for a period approximating the duration of processing for the other test group procedures and re-implanted without any significant modification. This process simulates real-world scenarios that are encountered when teeth are avulsed, gently cleaned, and stored in solution before re-implantation in a dental office setting (*n* = 2; one (1) maxillary and one (1) mandibular).

### Test Group 1 (Particulate Implant, T1)

As each tooth was extracted, a polyvinyl siloxane mold was created of its original form, and it the tooth was processed to dentin by grinding the entire tooth once the enamel was removed with a highspeed handpiece and diamond bur. The dentin was then processed following the KometaBio® (Smart Dentin Grinder®) machining and cleansing protocols and mixed with a marketed dental cement, Biodentine®, to form a composite material. This chemical cleansing protocol involved treating the ground dentin with a 0.5 M NaOH and 30% alcohol solution for 10 min and then two rinses in sterile phosphate-buffered saline (PBS) for 3 min before drying on a 140 °C hot plate for 5 min. The composite material was packed into a mold form that mimics the shape and composition of the original tooth. Once set, the implant was removed from the mold and placed in the extraction socket (*n* = 4; at least one (1) maxillary and one (1) mandibular per animal).

### Test Group 2 (Shell Implant Alone, T2)

As each tooth was extracted, it was processed to dentin by once again making a mold and then grinding just the internal portion of the tooth. The dentin was processed by subjecting the dentin shell to the chemical cleansing protocol described in Test Group 1 and then air-dried and mixed with Biodentine® cement to form a composite material. The shell implant was prepared by filling the residual shell with the cement material while inside the mold. Once set, the implant was removed from the mold and placed in the extraction socket (*n* = 2; at least one (1) maxillary and one (1) mandibular per animal).

### Test Group 3 (Shell Implant+Emdogain^®^, T3)

The same process was repeated for Test Group 2. Additionally, Emdogain® (EMD), an enamel matrix-derived regenerative material, was applied to the root surface of the tooth and the extraction socket before the implant was placed. The implant then was placed in the extraction socket. (*n* = 3; at least one (1) maxillary and one (1) mandibular per animal).

**Fig. 1:**
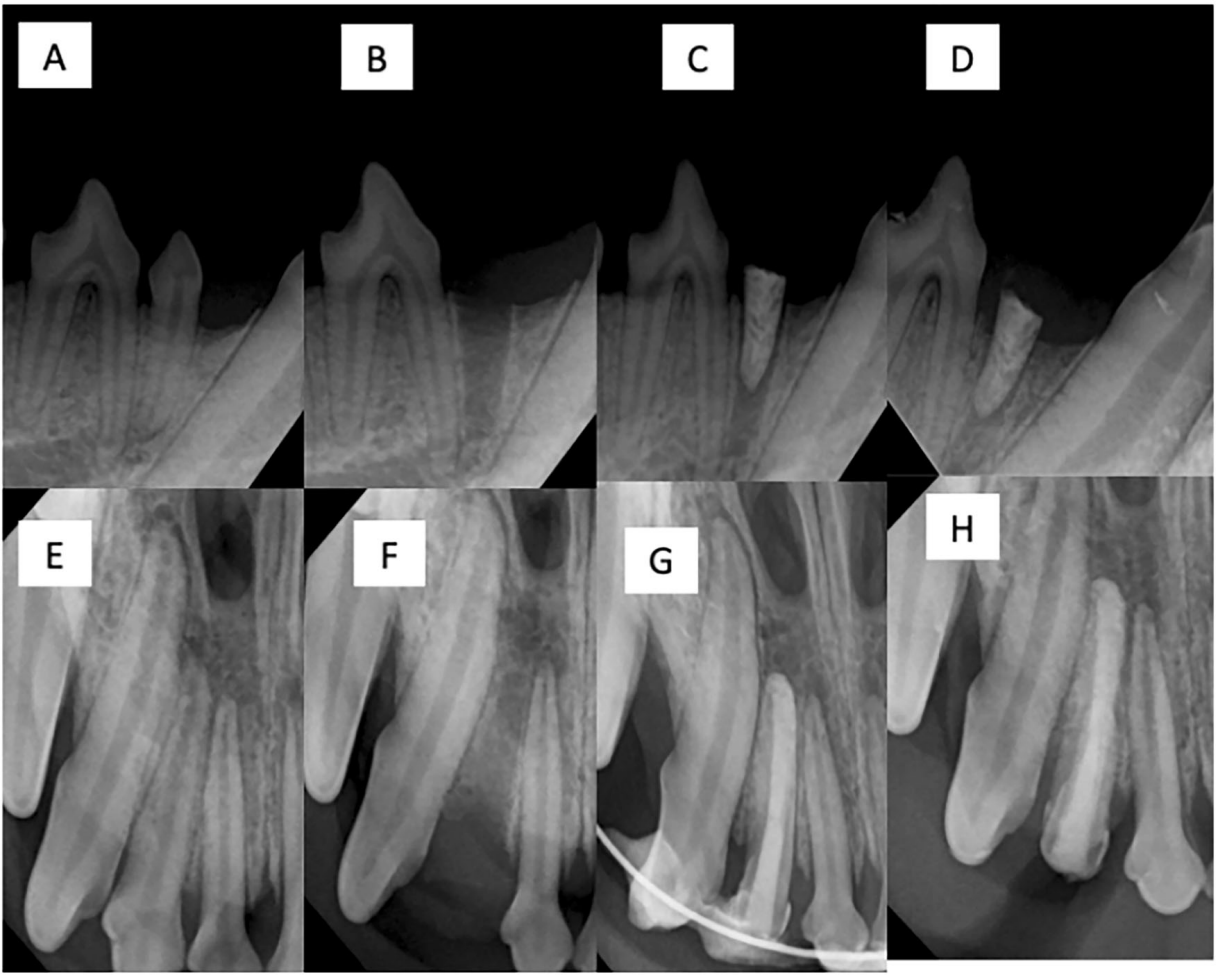
Animal radiographic images: **A**–**D** include radiographis for a mandibular right first premolar. **A** Day pre-extraction. **B** Day 0 post-extraction. **C** Day post-implantation. **D** Termination. **E**–**H** Include radiographis for a maxillary right lateral incisor. **E** Day 0 pre-extraction. **F** Day 0 post-extraction. **G** Day post-implantation. **H** Termination

In all groups, the implants were splinted to the adjacent teeth by using 0.018 inch round stainless-steel orthodontic wire, which was bonded to the coronal portion of the teeth and implants by using flowable composite resin after a 30 s etch, followed by application of a bonding agent, which was light-polymerized for 60 s intraorally. Once the resin fully polymerized, post-implantation periapical radiographs were made. One implant in Test Group 1 was left intentionally partially implanted to observe the effect of periapical bone fill surrounding these implants in clinical scenarios where a gap between the implant and socket wall is present (as seen in Fig. 1). Postoperative antibiotics were given for 7 days, analgesics were administered for up to 5 days, and the dogs were kept on a liquid diet for the first 3 days, followed by a soft food diet after that. At four weeks after implantation, general anesthesia was induced again to allow for clinical re-evaluation and periapical radiographs. Animals were euthanized and block sections of the mandibular and maxillary test sites were taken and placed in 10% neutral buffered formalin.

### MicroCT

Upon receipt of the fixed tissues, micro-CT scans of the entire implant site were performed at 18-micron resolution on a μCT 80 (Scanco, Switzerland) scanner. Following scanning, representative 2D slices were imaged in the transverse and sagittal plane at the center of each implant. The scans were trimmed to include only the implant and peri-implant tissues. The scan was then segmented, based on the density of Biodentine® and tooth, into two separate volumes. The measured volume (mm^3^) of each scan was determined. Identification of peri-implant mineral deposits (apical and lateral), peri-implant osseointegration (ankyloses) – apical and lateral vs. peri-implant periodontal ligament regeneration assessment and homogeneity of the implant was assessed qualitatively. This qualitative analysis was done by using 2D slices, 3D models, and representative images generated for the two different testing groups. The images were also used to quantitate Biodentine® volume (BV), the total volume of the region of interest (TV), and the ratio of the two (BV/TV). Any elicited responses to the implant were used in the comparison between the two groups.

### Pathology

Treatment sites were histologically processed at North American Science Associates Inc. (NAMSA), 6750 Wales Road, Northwood, Ohio 43619, and the resulting slides were sent to Alizée Pathology, Inc., 20 Frederick Road, Thurmont, MD 21788 for pathological evaluation. The implant sites were processed by using the EXACT® method (embedded in plastic, sectioned, surface edged, and stained). Pathological evaluation of tissue response to the Particulate Implant (Test Group 1, T1), Shell Implant Alone (Test Group 2, T2), Shell Implant + Emdogain® (Test Group 3, T3), or Not Treated (Control Group, C1) was completed by a board-certified veterinary pathologist, via light microscopy. Tissue sections stained with H&E were used to evaluate the cellular response following the scoring criteria described in the International Organization for Standardization (ISO) 10993-6, Part 61, as well as root resorption. Also, tissue responses including, but not limited to, lamellar and woven bone regeneration, residual implant material, and periodontal ligament fibers were evaluated in tissue sections stained with MGT.

## Results

### Radiographic/Clinical

**Fig. 2:**
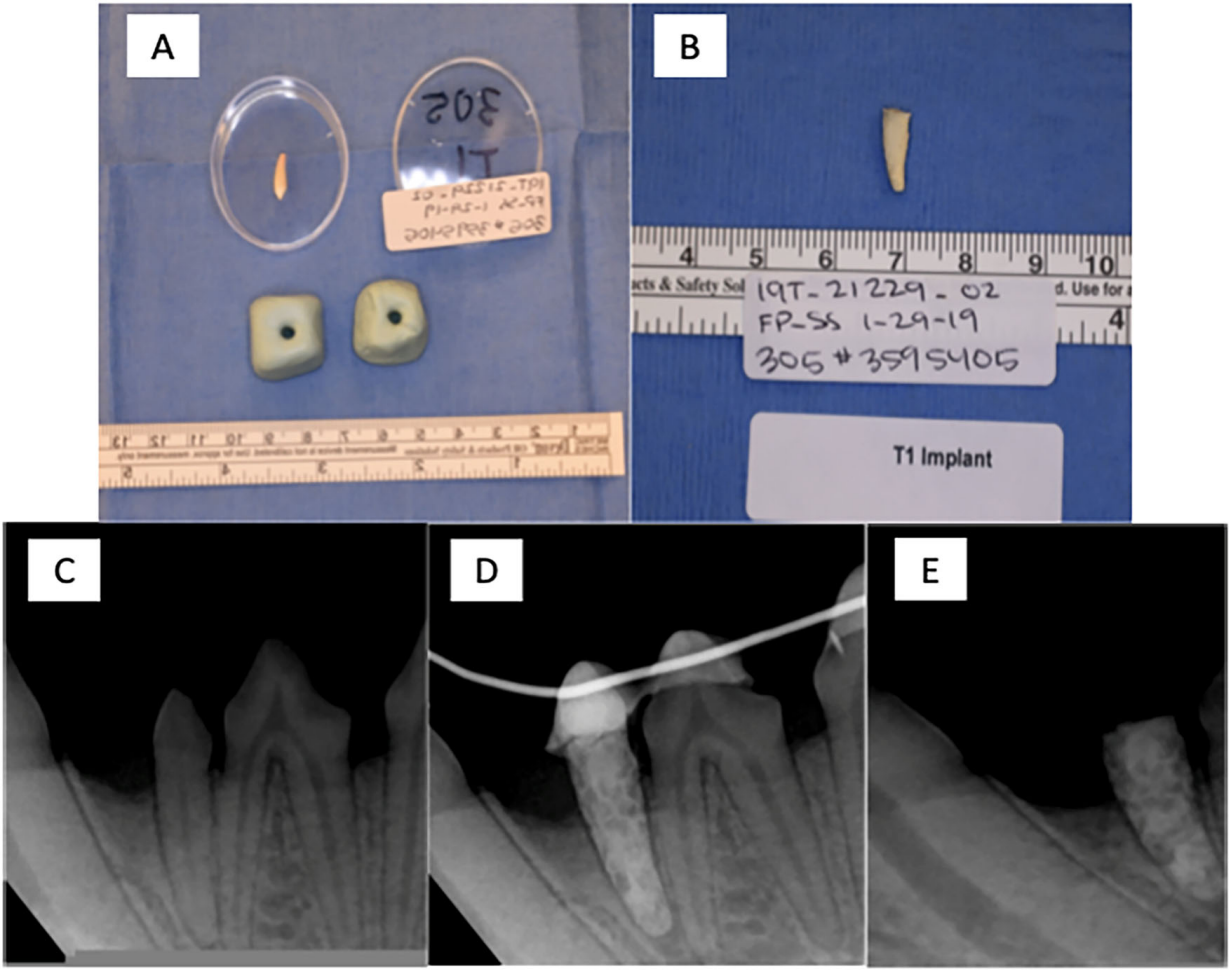
Radiographs of dentin/cement composite implants at 4-weeks post implantation. **A** Tooth and mold preparation. **B** Chairside implant fabrication T1 implant. **C** Natural tooth (mandibular left first premolar) before extraction – pre-extraction. **D** Implant immediately post-implantation and splinting-post implant. **E** Implant at 4 weeks post op after splint removal. Termination

**Fig. 3:**
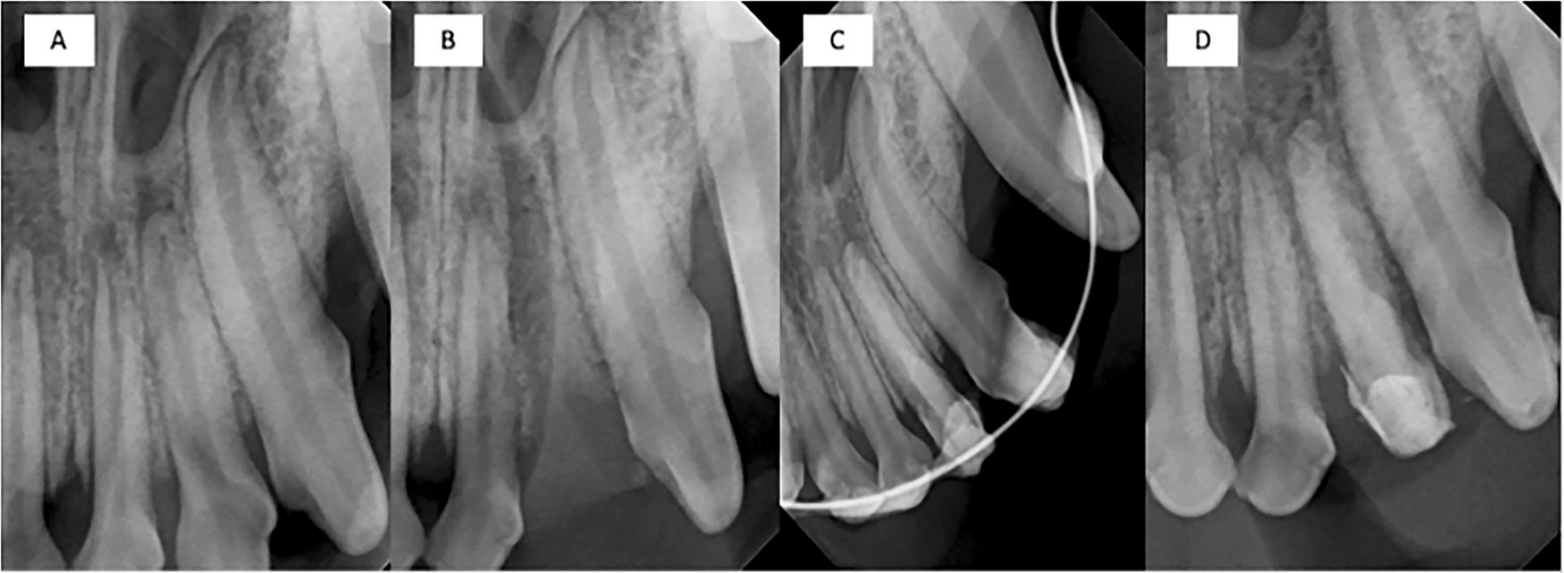
Radiographic images: figure includes radiographis for a maxillary left lateral incisor. **A** Day 0 pre-extraction. **B** Day 0 post-extraction. **C** Day post implantation. **D** Termination

One of the crucial questions relates to what happens at the implant to periodontal tissue interface during physiologic healing. The focus is on assessing the rate and quality of healing in an in vivo model. As shown in Fig. 2, the results of our in vivo pilot study in beagle dogs revealed that not only are these implants biocompatible, but their integration in the alveolus is likely secondary to a ligamentous attachment suggested by the findings of no implant mobility with conservation of a periodontal ligament space after splint removal at 4 weeks. Additionally, as observed in the figures below, all test groups including the controls showed no signs of infection or bone loss after the 4-week re-evaluation (Fig. 3).

### Micro-computed tomography

**Fig. 4:**
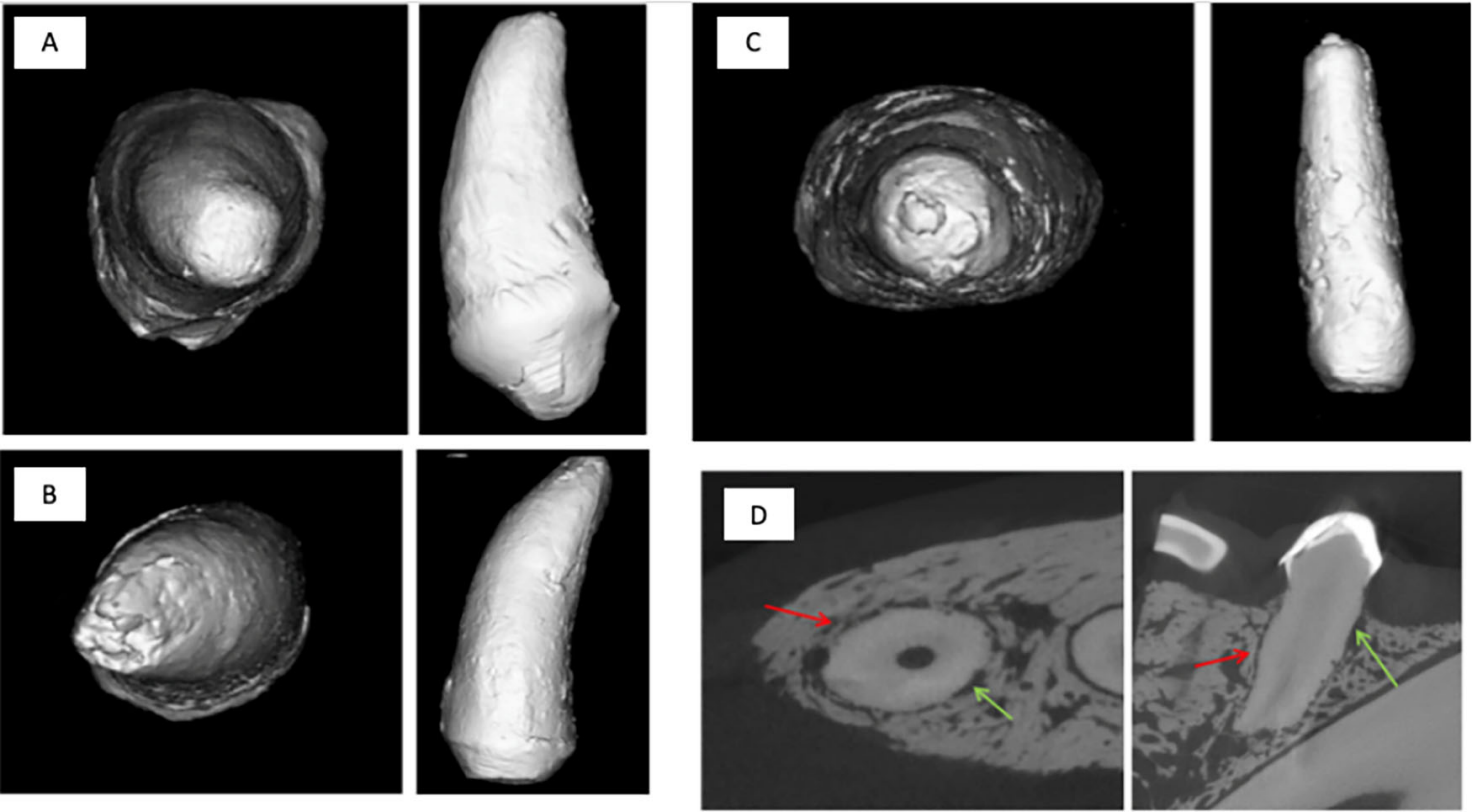
3D rendering of T0 control groups and representative 2D microCT. **A** T0 of an extracted tooth. **B** T1 implant group. **C** T2/T3 implant group. **D** Slice at about the coronal/transverse midline of an implant control specimen. Red arrows indicate regions where calcified bony tissue has appeared to be ingrown into the periodontal ligament space, and green arrows indicate area where the space appears to be composed of soft tissue or gaps in the calcified bony tissue

Representative 2D microCT slices for all specimens can be seen in Fig. 4 slice at about the coronal/transverse midline of an implant control group specimen (left mandibular first premolar, 305, animal 3580416). The implant control group was an extracted tooth that was scaled and then re-implanted. Red arrows indicate regions where calcified bony tissue has appeared to be ingrown into the periodontal ligament space, and green arrows indicate areas where the space appears to be composed of soft tissue or gaps in the calcified bony tissue.

### Histology tissue response

Both implant and non-implant (i.e., procedural) related inflammation was assessed as follows in Table 1. In addition, the corresponding inflammatory cell types (polymorphonuclear cells [neutrophils/eosinophils], lymphocytes, plasma cells, macrophages, and multinucleated giant cells) were assessed as follows:

**Table 1:** Implant and non-implant-related inflammatory assessment

Parameter	Control	T1	T2	T3
Mean ± SD implant-related inflammatory cell types				
Implant inflammation	–	1.0 ± 0.0	1.0 ± 0.0	0.8 ± 0.3
Polymorphonuclear (Neutrophils/Eosinophils)	–	0.3 ± 0.3	0.3 ± 0.4	0.0 ± 0.0
Lymphocytes	–	0.8 ± 0.3	0.8 ± 0.4	0.5 ± 0.5
Plasma cells	–	0.5 ± 0.0	0.0 ± 0.0	0.2 ± 0.3
Macrophages	–	1.0 ± 0.0	1.0 ± 0.0	0.8 ± 0.3
Multinucleated giant cells	–	0.6 ± 0.3	0.5 ± 0.0	0.3 ± 0.6
Mean ± SD non-implant-related inflammatory cell types				
Overall inflammation	1.0 ± 0.0	1.6 ± 0.5	1.0 ± 0.0	1.0 ± 0.0
Polymorphonuclear (Neutrophils/Eosinophils)	1.0 ± 0.0	0.9 ± 0.5	0.8 ± 0.4	0.7 ± 0.3
Lymphocytes	1.0 ± 0.0	0.9 ± 0.3	1.0 ± 0.0	0.8 ± 0.3
Plasma cells	0.5 ± 0.7	0.8 ± 0.3	0.5 ± 0.7	0.8 ± 0.3
Macrophages	1.0 ± 0.0	1.5 ± 0.6	1.0 ± 0.0	1.0 ± 0.0
Multinucleated giant cells	0.0 ± 0.0	0.1 ± 0.3	0.0 ± 0.0	0.0 ± 0.0

Inflammation Scoring Matrix: 0 = Not present; 1 = Present but slight feature; 2 = Notable feature, mild; 3 = Prominent feature but is not overwhelming, moderate; 4 = Overwhelming feature, severe

Inflammatory Cell Types Scoring Matrix: 0 = Absent; 1 = Rare, 1–5 per high magnification field (hpf, 400x; giant cells = 1-2/hpf); 2 = 5–10/hpf (giant cells = 3-f/hpf); 3 = Heavy infiltrate (giant cell = numerous); 4 = Packed (giant cells = sheets)

As described in Table 1, implant-related inflammation (evaluated for Groups T1, T2 and T3) was on average similar among all Groups, with low numbers of infiltrates in all specimens evaluated. Implant-related inflammatory reaction was generally minimal and not interpreted to be adverse. Non-implant-related inflammation (i.e., procedural) was similar among all groups (Control, T1, T2, T3 and T4). Inflammation was as expected for an oral implant model and was not interpreted to be adverse.

**Fig. 5:**
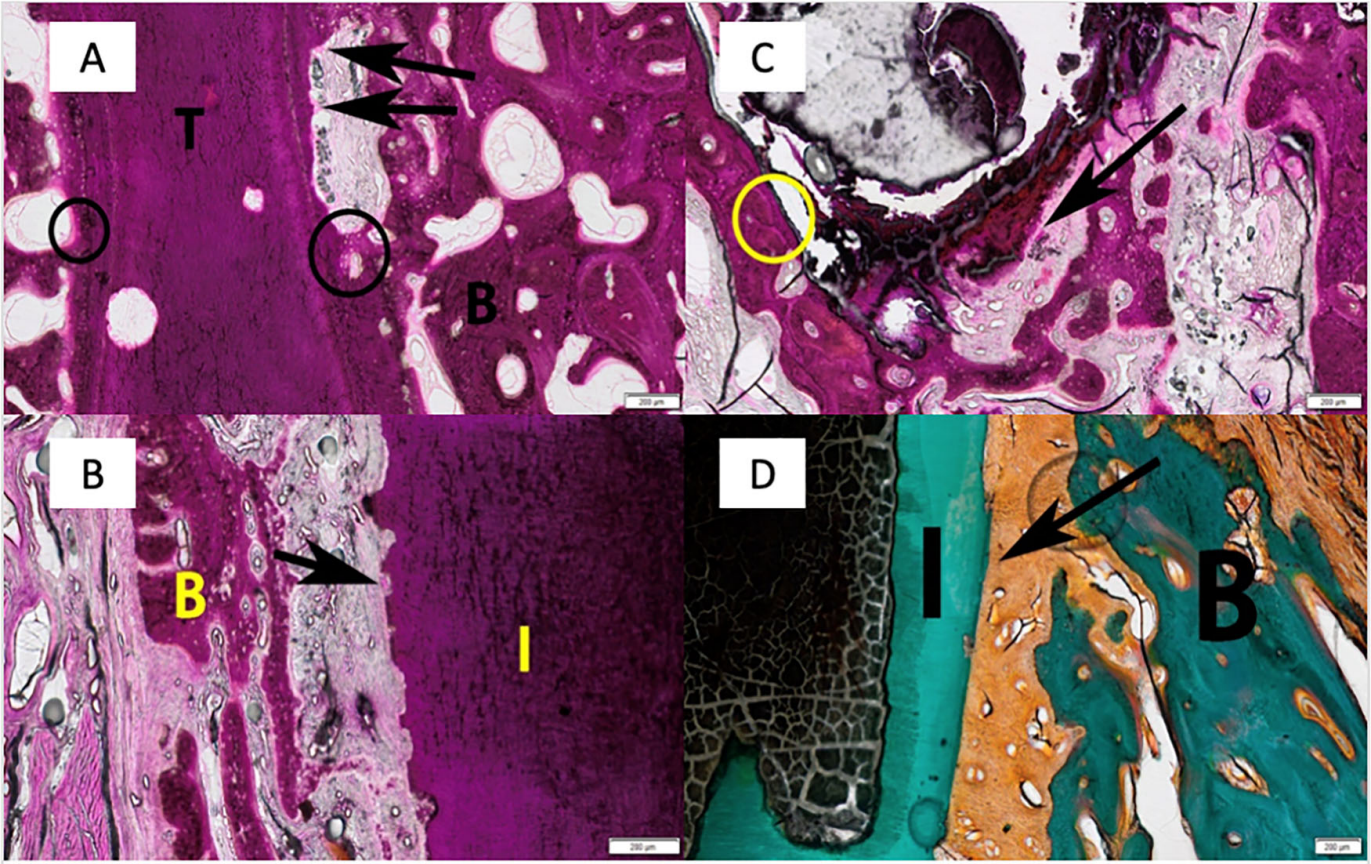
Root resorption: **A** Level 1. Control. Week 4. Note root resorption (black arrows) and direct apposition between bone and tooth (circles). B Bone, T Tooth. **B** Level 2. Particulate implant. Week 4. Note root resorption (black arrow0 and direct apposition between bone and implant (circle). B Bone, T Tooth. **C** Level 4. Shell Implant Alone. Week 4. Note root resorption (black arrow). B Bone, I Implant. **D** Level 2. Shell implant + Emdogain®. Week4. PDL fibers are shown (arrow). B Bone, I Implant

Tissue response evaluation looked at the parameters characterizing tissue reaction surrounding the implant sites and included necrosis, fibrosis, fatty infiltrates, neovascularization, hemorrhage, epithelialization, and epithelial hyperplasia, which is outlined in Tables 2 and 3. In general, fibrosis varied from a narrow band (Score 1) to a moderately thick band (Score 2) in all groups. There was no implant encapsulation in any of the evaluated specimens. Overall average fibrosis scores were highest in Group T3 (Shell Implant + Emdogain®), but fibrosis scores were not associated with decreased bone regeneration, as is apparent from the histologic slices in Fig. 5, or with decreased numbers of PDL fibers. Overall average neovascularization and epithelialization scores were as expected for this oral surgical model and biologically similar for all groups. Epithelial hyperplasia was overall minimal and as expected for this canine oral surgical model. There was no evidence of fatty infiltration, necrosis, infection, or suppurative or granulomatous inflammation in any of the specimens evaluated. Hemorrhage, when present, was focal, minimal, and non-adverse.

**Table 2:** Necrosis, fibrosis, fatty infiltrates, neovascularization, hemorrhage, epithelialization and epithelial hyperplasia assessment

Necrosis	0	Absent
	1	Minimal
	2	Mild
	3	Moderate
	4	Severe
Fibrosis	0	Absent
	1	Narrow Band
	2	Moderately thick band
	3	Thick band
	4	Extensive band
Fatty Infiltrates	0	Absent
	1	Minimal amount of fat associated with fibrosis
	2	Several layers of fat and fibrosis
	3	Elongated and broad accumulation of fat cells about the implant site
	4	Extensive fat completely surrounding the implant
Neovascularization	0	Absent
	1	Minimal capillary proliferation, focal, 1 to 3 buds
	2	Groups of 4 to 7 capillaries with supporting fibroblastic structures
	3	Broad band of capillaries with supporting structures
	4	Extensive band of capillaries with supporting fibroblastic structures
Hemorrhage, epithelialization, and epithelial hyperplasia	0	Not present
	1	Present but slight feature, minimal
	2	Notable feature, mild
	3	Prominent feature but is not overwhelming, moderate
	4	Overwhelming feature, severe

**Table 3:** Mean tissue response comparison to control

	Parameter	Control	T1	T2	T3
Mean ± SD Tissue	Necrosis	0.0 ± 0.0	0.0 ± 0.0	0.0 ± 0.0	0.0 ± 0.0
	Fibrosis	1.3 ± 0.4	1.3 ± 0.4	1.5 ± 0.7	1.8 ± 0.3
	Fatty Infiltrate	0.0 ± 0.0	0.0 ± 0.0	0.0 ± 0.0	0.0 ± 0.0
	Neovascularization	0.8 ± 0.4	1.1 ± 0.3	1.0 ± 0.0	1.2 ± 0.3
	Hemorrhage	0.0 ± 0.0	0.0 ± 0.0	0.3 ± 0.4	0.2 ± 0.3
	Epithelialization	3.8 ± 0.4	3.9 ± 0.3	4.0 ± 0.0	3.8 ± 0.3
	Epithelial Hyperplasia	1.3 ± 0.4	1.3 ± 0.5	1.0 ± 0.0	0.8 ± 0.3

*SD* Standard Deviation

Necrosis Scoring Matrix: **0** = Absent; **1** = Minimal; **2** = Mild; **3** = Moderate; **4** = Severe

Fibrosis Scoring Factor: **0** = Absent; **1** = Narrow band; **2** = Moderately thick band; **3** = Thick band; **4** = Extensive band

Fatty Infiltrate Scoring Matrix: **0** = Absent; **1** = Minimal amount of fat associated with fibrosis; **2** = Several layers of fat and fibrosis; **3** = Elongated and broad accumulation of fat cells about the implant site; **4** = Extensive fat completely surrounding the implant

Neovascularization Scoring Matrix: **0** = Absent; **1** = Minimal capillary proliferation; **2** = Groups of 4 to 7 capillaries with supporting fibroblastic structures; **3** = Broad band of capillaries with supporting structures; **4** = Extensive band of capillaries with supporting fibroblastic structures

Morphological Changes Scoring Matrix: **0** = Not present; **1** = Present but slight feature, minimal; **2** = Notable feature, mild; **3** = Prominent feature but is not overwhelming, moderate; **4** = Overwhelming feature, severe

## Discussion

This study examined the first application of combining dentin and CSC for use as a load-bearing and biocompatible dental implant. In regard to the antibacterial effects of CSC and MTA® in particular, several investigations tested and reported on the antibacterial effects of mineral trioxide aggregate on a range of microorganisms [12–14]. In one study comparing the antimicrobial effects of root-end filling materials against S. aureus, E. faecalis, and P. aeruginosa the authors reporated that IRM® and Gray MTA® demonstrated a more significant antibacterial effect compared with the other tested biomaterials [15]. Additionally, other studies revealed that Gray MTA® [16] and White MTA® [17] also have an antifungal effect, including on C. albicans. It is believed that this antimicrobial effect of MTA® may be because of its high pH or because of ion release from the MTA® into surrounding local environments [18]. Based on the wealth of information available about these materials, it appears that MTA® is a bioactive material with the inherent ability to create an ideal wound healing environment. As soon as the material is implanted, the cement matrix forms calcium hydroxide which releases calcium ions and aids in cell attachment and proliferation [19–25]. Calcium hydroxide in the cement matrix raises the pH of the material’s surface to an alkaline environment [16, 26] and increases cytokine production [27, 28] that enhances differentiation and migration of osteopromotive cell types [29]. Calcium hydroxide also forms a carbonated apatite layer on the MTA surface, thereby creating a biologic seal that relates specifically to the bioactivity of the materials [30]. Another study tested the effects of ground dentin powder in combination with either Bioaggregate (BA) or MTA powder compared to the cement materials alone. It observed that the addition of equal amounts of dentin powder to the suspension of either BA or MTA powder, which were both prepared as fresh and set materials, resulted in faster elimination of E. faecalis in an in vitro study [31]. This interesting and important finding allows us to transition to the next important component of our study. The second key component of our approach is the processing of autologous dentin for reuse in the oral cavity. This concept was initially developed to serve as an additional source of bone void filler material for alveolar ridge and socket preservation following tooth extraction. We used technology that was already introduced into the market by Binderman et al., known as the Smart Dentin Grinder (manufactured by Kometabio, Inc.) for the application of processing extracted teeth into autologous particulate dentin autograft material [32]. Their process, which has been documented extensively in the literature, involves extracting human teeth, grinding and sorting the material, cleansing the particulate dentin, and grafting these particles back into the socket as an alternative to the use of traditional particulate bone graft for socket preservation [32]. The practice of tooth reimplantation has been documented since the beginning of the 18th century, and since then, a vast amount of information has been collected about the factors contributing to its high success rates under certain conditions. The most common factors influencing these outcomes involve the length of extraoral time before reimplantation, the amount of trauma to the periodontal structures, duration of splinting, and more recently, the addition of adjunctive treatments such as the application of growth factors like enamel matrix derived proteins (EMD) and platelet-derived growth factor (PDGF) to support periodontal regeneration [33–35]. Studies have also outlined the viability and regenerative capacity of periodontal ligament fibroblasts that are present and collected immediately after tooth extraction [36].

The rationale for our alternative approach to tooth replacement and the anticipated mechanism of healing resembles reinsertion of avulsed teeth as a result of trauma far closer than it does osseointegration secondary to osteotomy preparation and titanium implant placement. This difference is important because the majority of peri-implant diseases are the result of the differences in orientation and quality of connective tissue fibers within the gingival cuff and missing down the surface of the titanium that leave implants susceptible to significant bone loss. While it is true that titanium will always be stronger than natural teeth, there is still a substantial biochemical and mechanical mismatch between this inert metal alloy and surrounding tissues in the body, which is the reason for many of the observed failures that have motivated our research. Reinsertion in less than an hour and proper stabilization of the implant during the healing period is essential for success, as is documented in the literature. The justification for choosing the materials (autologous dentin and a CSC) is that they naturally complement each other in their biological and physical properties and have both demonstrated the capacity to develop new periodontal attachments on their previously denuded surfaces. These new attachments include the generation of new cementum, periodontal ligament fibers, and alveolar bone. The main advantages of periodontal regeneration versus ankylosis or “fusion” of implants to the jawbone include the maintenance of proprioception, which is recorded within the periodontal ligament space, and a cushioning effect, thereby reducing stress transfer to the surrounding structures.

The results of the current study suggest that this novel material can integrate successfully into the alveolar processes in a canine model. The parameters evaluated to examine the tissue response of these implants included clinical, radiographic, and histologic assessment. The data suggest no significant adverse effects when evaluating these parameters in test groups compared to controls. Within the limited number of test specimens that were examined in this pilot study, the outcomes suggest a promising personalized implantation method that may pave the way for future research into the concept of customized immediate dental implants. This novel implant method and material may also allow for a safer and more affordable procedure that can be performed by a broader range of providers. The reason for the choice is because of the intraoperative simplicity of this technique, which was specially designed for clinicians with limited conventional implant surgical training and equipment to provide another therapeutic option for their patients. Further advancements in this concept may someday address the significant issue of access to care and tooth replacement, particularly in developing nations or rural areas. It also has potential applications in multiple age groups, especially patients between the ages of 6–21 years of age (a subset of the population that previously was unable to receive fixed implants because of complications of titanium implants and hindered craniofacial growth) [37], numerous clinical scenarios because of the versatility of implant design can be applied globally because of the nature and availability of the materials, rapid chairside fabrication methods, and the relatively inexpensive cost of materials, equipment, and training. The goal of this research is to develop a system that is reproducible and may provide an opportunity for tooth replacement in areas of the world that have limited resources, access to care, and currently no means of tooth replacement. We hope that this technique could dramatically reduce the high incidence of edentulism globally and mitigate the detrimental medical effects of malnutrition and untreated dental infections [38].

## Conclusion

Based on the limitations of this study, the results revealed that not only do these materials demonstrate high biocompatibility, but their integration in the alveolus is likely secondary to a partial ligamentous attachment suggested by the findings of no implant mobility with the conservation of a periodontal ligament space after splint removal at 4 weeks. The histological evaluation suggests favorable integration of all implants with the surrounding peri-implant tissues and minimal inflammation present compared to controls. The current investigation may open new avenues into the use of CSC materials for the fabrication of custom dental implants. Further research into the biomechanical properties of this novel composite is necessary to develop the optimal material composition for use as a load-bearing dental implant.
